# Theoretical study of superionic phase transition in Li_2_S

**DOI:** 10.1038/s41598-017-05775-2

**Published:** 2017-07-19

**Authors:** Sara Panahian Jand, Qian Zhang, Payam Kaghazchi

**Affiliations:** Institut für Chemie und Biochemie, Freie Universität Berlin, Takustr, 3, 14195 Berlin, Germany

## Abstract

We have studied temperature-induced superionic phase transition in Li_2_S, which is one of the most promising Li-S battery cathode material. Concentration of ionic carriers at low and high temperature was evaluated from thermodynamics of defects (using density functional theory) and detailed balance condition (using *ab initio* molecular dynamics (AIMD)), respectively. Diffusion coefficients were also obtained using AIMD simulations. Calculated ionic conductivity shows that superionic phase transition occurs at *T* = 900 K, which is in agreement with reported experimental values. The superionic behavior of Li_2_S is found to be due to thermodynamic reason (i.e. a large concentration of disordered defects).

## Introduction

Ionic conductivity of electrode and electrolyte materials is an important parameter for the operation of Li-based batteries. Mechanism and rate of Li diffusion through Li-based battery materials have therefore been studied extensively^1–4^. In addition, different strategies have been proposed to increase carrier concentration and mobility (i.e. improving Li ion conductivity) in solid state materials^2, 5, 6^. Moreover, superionic compounds are widely investigated because of their fundamental interests and potential applications in solid-state batteries, fuel cells, and gas sensors^7–12^. These ionic crystals have high ionic conductivity at temperatures below the melting point. Above the temperature of superionic phase transition one of the ion sublattices becomes disordered leading to a high ionic conductivity. Extensive experimental and theoretical studies have been carried out to understand the structure and diffusion behaviour of superionic compounds. Experimental neutron diffraction^13–16^ and theoretical molecular dynamics (MD)^17–20^ are the most commonly used techniques to investigate these materials. Li-based compounds are of special interests due to their applications in Li-based batteries. In particular, Li_2_S is one of the most promising Li-S battery cathode materials. Ionic conductivity of Li_2_S is a key factor determining the performance of Li-S batteries. Li_2_S is also interesting from scientific point of view as it is a supersonic conductor at temperatures higher than ≈900 K ^21^. The ionic conductivity of Li_2_S has been measured to be 1.27 × 10^−1^ S/cm at 1170 K ^22^. The quasielastic neutron scattering study by Altorfer *et al*.^22^ proposed two models for Li transport in the superionic phase of Li_2_S: (i) Li vacancy jumps between regular Li sites and (ii) Li jumps between regular Li and interstitial defective sites. In spite of the importance of Li-S batteries, temperature-dependent Li ion conductivity in Li_2_S has not been theoretically studied so far. In this work, we combine *ab initio* molecular dynamics (AIMD) and density functional theory (DFT) as well as thermodynamic and kinetic considerations to calculate Li ion conductivity as function of temperature and apply this approach to study the mechanism of superionic phase transition in Li_2_S.

**Figure 1:**
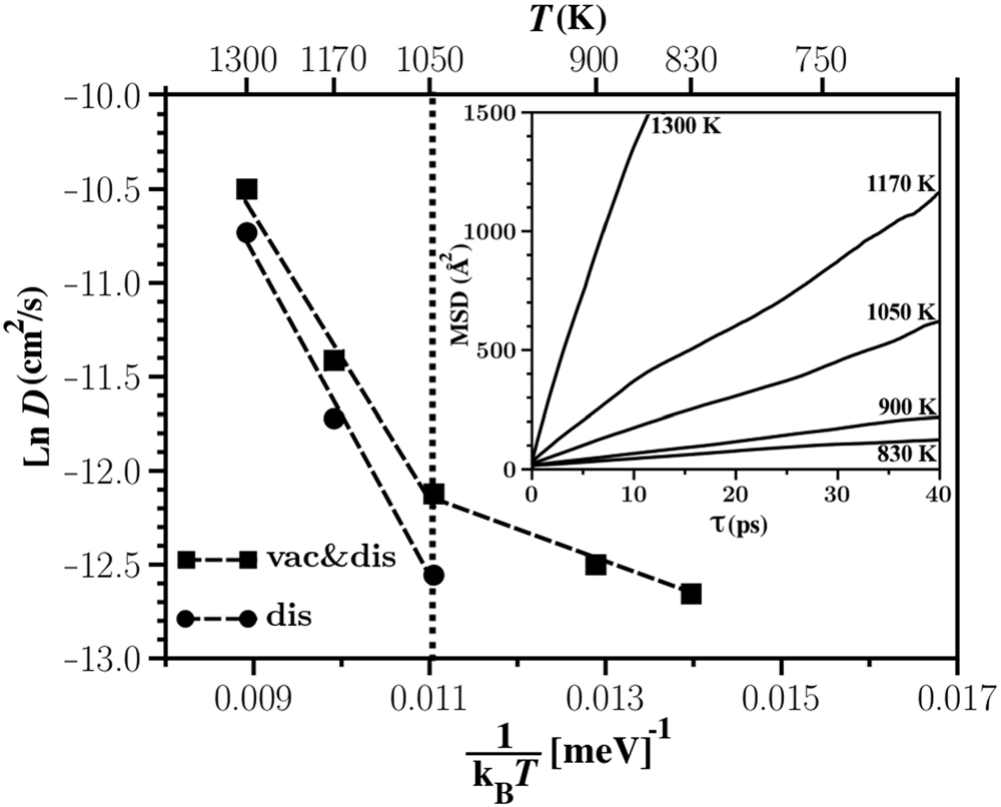
Arrhenius plot of diffusion coefficients in Li_2_S. Diffusion coefficient in the pristine structure is shown by *D*_dis_ since in this case Li ion migration takes place via temperature–induced disorder (interstitial Li). Li diffusion in Li_2_S with a single vacancy is illustrated by vac&dis as charge carriers are both Li vacancy and disorder (interstitial Li). The inset presents total mean square displacements (MSD) versus lag time calculated for 40 ps AIMD simulations.

To evaluate diffusion coefficients, 50 ps AIMD simulations (see Method part for further details) were carried out for pristine Li_2_S and Li_2_S with a single vacancy at temperatures *T* = 300, 600, 750, 830, 900, 1050, 1170, and 1300 K. The thermalization was achieved within 10 ps and the rest 40 ps were used for structure sampling. Note that it is very important to perform the AIMD simulation without considering any symmetry constraint. Total mean square displacements of Li ions as function of lag time (MSD(*τ*)) for Li_2_S (modelled using 2 × 2 × 2 unit cells) with a single Li vacancy at different temperatures are illustrated in Fig. 1. Only few number of Li vacancy hoppings were observed for *T* = 300, 600, and 750 K. Therefore we have not considered these temperatures in Fig. 1. For higher temperatures, we have observed more than 32 jumps in Li_2_S (modelled with a 2 × 2 × 2 unit cell) during 40 ps AIMD simulation which is expected to be sufficient for our analysis. Calculated diffusion coefficients of Li in Li_2_S with one Li vacancy using the Einstein relation (*D*) indicate a change in slope at 1050 K (see Fig. 1), showing that Li diffusion occurs via a mechanism distinct from the Li vacancy hopping at high temperatures.

**Figure 2:**
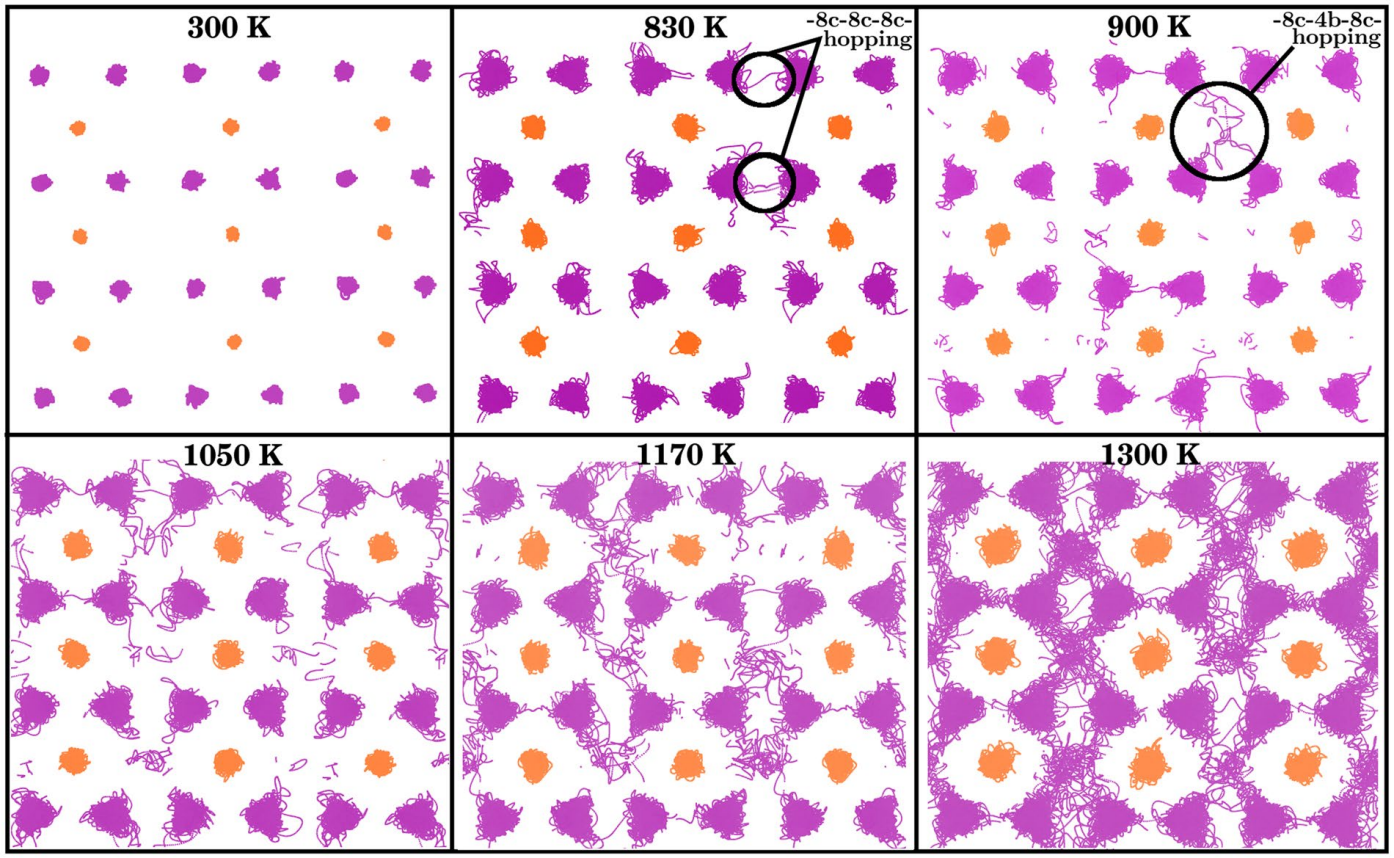
Visited Li and S ion positions in the {$1\bar{1}0$} planes of Li_2_S within slices of 1.7 Å thickness during 40 ps of AIMD simulations at 300 K, 830 K, 900 K, 1050 K, 1170 K and 1300 K. The visited positions by Li and S ions are in purple and gold, respectively.

To uncover the mechanism of Li diffusion at high temperatures, we extracted visited positions of Li and S ions within the {$1\bar{1}0$} planes of Li_2_S with a single Li vacancy at different temperatures (Fig. 2). It is found that Li transport occurs mainly via Li vacancy hopping between regular Li sites (so called 8c sites) at low temperatures such as *T* = 830 K. At higher temperatures anharmonic elongation in Li ion positions appear. Although Li transport still takes place mainly via Li vacancy hopping between 8c sites, there are few Li jumps between 8c and interstitial defective sites (so called 4b sites) at *T* = 900 K. At *T* = 1050 K, Li transport is due to both –8c–8c–8c– and –8c–4b–8c– mechanisms. However, Li diffusion at 1170 K and 1300 K is mainly along the latter pathway. The only possible mechanism for Li migration at low temperatures is the vacancy hopping, and *D*_vac_ is calculated directly from the Einstein relation. *D*_vac_ for temperatures higher than 1050 K is obtained by extrapolating the low temperature values of *D*_vac_ (see Fig. 1). At 1170 K, the value of D for diffusion of Li along –8c–4b–8c– (*D*_dis_) is larger than the value of D for diffusion of Li along –8c–8c–8c– (*D*_vac_). Li diffusion takes place only through –8c–4b–8c– pathway in pristine structure (*D*_pris_ = *D*_dis_). For this reason, diffusion coefficient in pristine Li_2_S (modelled using 2 × 2 × 2 unit cells) *D*_pris_ at 1170 K and 1300 K is close to that in Li_2_S with a single vacancy. The calculated value of *D*_vac&dis_ = 1.03 × 10^−5^ cm^2^/s at 1170 K (superionic regime), which includes diffusion of Li along both –8c–8c–8c– and –8c–4b–8c– pathways, is in agreement with the experimental value of 1.39 × 10^−5^ cm^2^/s^22^. However, at this temperature the value of *D* for diffusion of Li along –8c–4b–8c– (*D*_dis_) is only 1.2 times larger than the value of *D* for diffusion of Li along –8c–8c–8c– (i.e. extrapolated value of *D*_vac_ from the low temperature regime). Therefore, the superionic behavior of Li_2_S is not due to kinetic reason.

To determine the most probable defect types and their concentrations (thermodynamic factor of ionic conductivity) in poor-ionic conductor phase, we need to obtain the formation energy of defects (${\rm{\Delta }}{E}_{{\rm{d}}}^{i,q}$). Recently, we have calculated ${\rm{\Delta }}{E}_{{\rm{d}}}^{i,q}$ for a variety of possible defects in poor ionic conductor phase of Li_2_S^23^ and found that charged interstitial Li (Li^+^) and Li vacancy (${{\rm{V}}}_{{\rm{Li}}}^{-}$) are the most favorable ionic charge carriers^23^. However, we have recently found that the values of ${\rm{\Delta }}{E}_{{\rm{d}}}^{i,q}$ depend strongly on several computational parameters such as unit cell size and the value of dielectric constant ($\epsilon$) that is used to correct the finite-cell size effect of charged defects and align the electrostatic potentials of defective and pristine supercells (Δ*E*). In this work, we recalculate ${\rm{\Delta }}{E}_{{\rm{d}}}^{i,q}$ using optimal parameters. Defect formation energies are calculated by

1 $${\rm{\Delta }}{E}_{{\rm{d}}}^{i,q}={E}_{{\rm{tot}}}^{i,q}-{E}_{{\rm{tot}}}^{{{\rm{Li}}}_{2}{\rm{S}}}+\sum _{i}{n}_{i}{\mu }_{{\rm{i}}}+q({\epsilon }_{{\rm{F}}}+{\epsilon }_{{\rm{VBM}}})+{\rm{\Delta }}E\mathrm{.}$$

Here, ${E}_{{\rm{tot}}}^{i,q}$ and ${E}_{{\rm{tot}}}^{{{\rm{Li}}}_{2}{\rm{S}}}$ are the total energies of defective and pristine Li_2_S, which have been calculated using DFT calculations (see Method part for further details). *n*_*i*_ and *μ*_*i*_ are the number and chemical potential of defects (Li or S). In our previous work, we used the electronic $\epsilon$ (${\epsilon }_{{\rm{el}}}$) calculated by density functional perturbation theory (DFPT)^23^. The value of ${\epsilon }_{{\rm{el}}}$ is generally smaller than the experimental value of static (low-frequency) $\epsilon$ (${\epsilon }_{{\rm{s}}}$). The value of ${\epsilon }_{{\rm{s}}}$ for Li_2_S at room temperature has been estimated by Yang *et al*.^24^ to be around 10, which is almost 3 times larger than our ${\epsilon }_{{\rm{el}}}$ value obtained from the DFPT method. In the present work, we use the value of ${\epsilon }_{{\rm{s}}}\approx 10$ to calculate Δ*E*. Moreover, instead of using a 2 × 2 × 2 unit cell which was applied in our previous work^23^, we use a 3 × 3 × 3 unit cell to calculate the total energies. *μ*_Li_ is considered to be smaller than the total energy per atom of bulk metal Li (${E}_{{\rm{tot}}}^{{\rm{Li}}}$). Moreover, it can not be smaller than a *μ*_Li_ value at which Li_2_S decomposes. By defining ${\rm{\Delta }}{\mu }_{{\rm{Li}}}$ to be ${\mu }_{{\rm{Li}}}-{E}_{{\rm{tot}}}^{{\rm{Li}}}$ the permitted range of Δ*μ*_Li_ is between 0 and half of the Gibbs energy of formation of Li_2_S.

To calculate ${\rm{\Delta }}{E}_{{\rm{d}}}^{i,q}$ and ${\epsilon }_{{\rm{F}}}$ as function of Δ*μ*_Li_, we consider the requirement of charge neutrality

2 $$\mathop{\underbrace{{\int }_{{\rm{CBM}}}^{\infty }D(\epsilon )f(\epsilon ,{\epsilon }_{{\rm{F}}})d\varepsilon }}\limits_{{n}_{{\rm{e}}}}-\mathop{\underbrace{{\int }_{-\infty }^{{\rm{VBM}}}D(\epsilon )[1-f(\epsilon ,{\epsilon }_{{\rm{F}}})]d\epsilon }}\limits_{{n}_{{\rm{h}}}}=\sum _{i}{q}_{i}\mathop{\underbrace{{n}_{i}^{0}\exp (-\frac{{\rm{\Delta }}{E}_{{\rm{d}}}^{i,q}}{{k}_{B}T})}}\limits_{{n}_{i}}\mathrm{.}$$

In this equation, *D*($\epsilon$) is the density of states, *f*(${\epsilon }$, ${\epsilon }_{{\rm{F}}}$) is the Fermi-Dirac distribution, *q*_*i*_ is the charge state of defect *i*. *n*_e_, *n*_h_, and *n*_i_ are the concentration of electrons, holes, and defects of type *i*, while ${n}_{i}^{0}$ is the maximum possible concentration of defects of type *i* per unit volume. Since eqs (1) and (2) are self-consistent, ${\rm{\Delta }}{E}_{{\rm{d}}}^{i,q}$ and ${\epsilon }_{F}$ are calculated iteratively.

**Figure 3:**
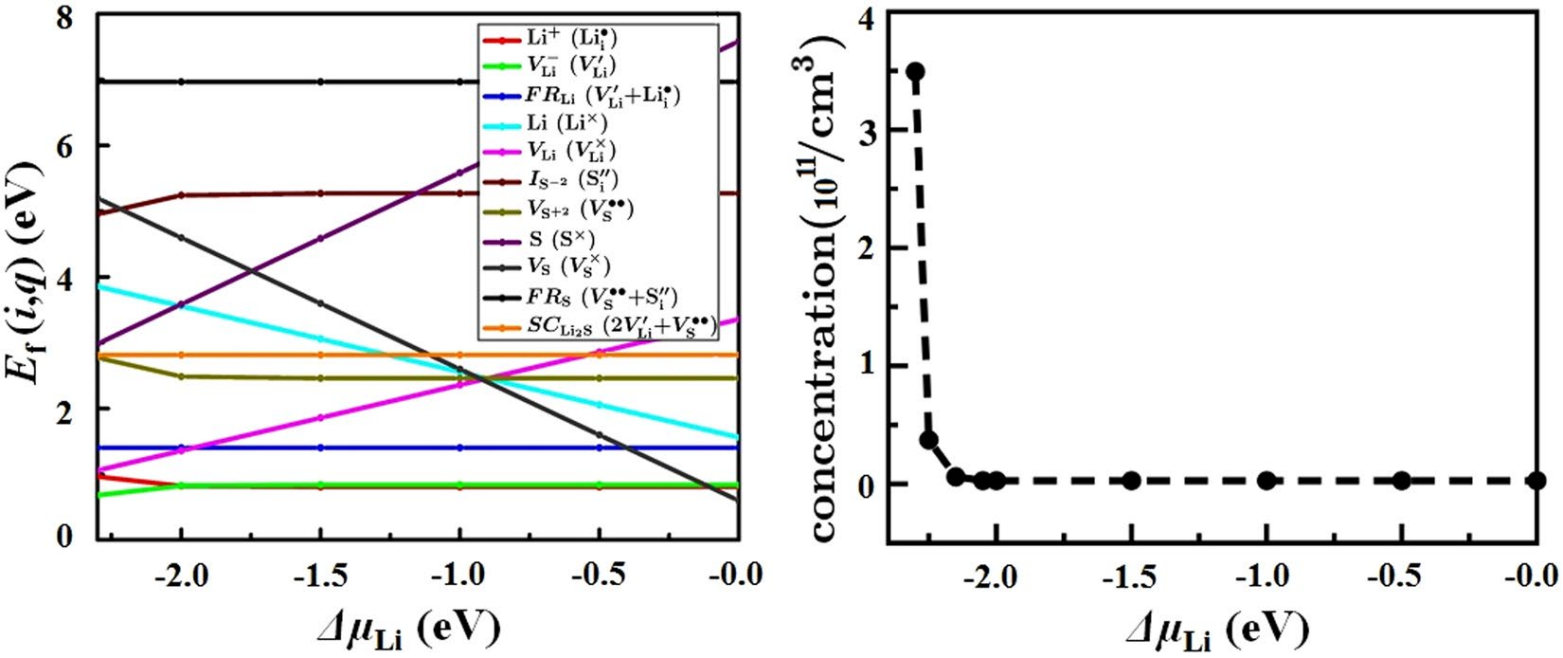
(left) Formation energies of different types of defects in bulk Li_2_S as function of Δ*μ*_Li_ (chemical potential of Li). Kroger–Vink representations are given in parentheses. (right) Concentration of Li vacancy as function of Δ*μ*_Li_.

Figure 3 shows that for −2.0 eV < Δ*μ*_Li_ ≤ 0 eV the formation energy of Li^−^ vacancy (${\rm{\Delta }}{E}_{{\rm{d}}}^{{V}_{{\rm{Li}}}^{-}}$) is 0.80 eV, which is 0.22 eV lower than the ${\rm{\Delta }}{E}_{{\rm{d}}}^{{V}_{{\rm{Li}}}^{-}}$ value calculated for ${\epsilon }_{{\rm{e}}}\approx 3.6$ and 2 × 2 × 2 unit cells. The formation energy of Li^+^ interstitial (${\rm{\Delta }}{E}_{{\rm{d}}}^{{{\rm{Li}}}^{+}}$) is very similar to ${\rm{\Delta }}{E}_{{\rm{d}}}^{{V}_{{\rm{Li}}}^{-}}$. Therefore, for −2.0 eV < Δ*μ*_Li_ ≤ 0 eV ionic transport in Li_2_S occurs via formation of Frenkel ${V}_{{\rm{Li}}}^{-}$ + Li^+^ pairs and their diffusion.

Figure 3 also demonstrates that for −2.3 eV ≤ Δ*μ*_Li_ ≤ −2.0 eV the value of ${\rm{\Delta }}{E}_{{\rm{d}}}^{{V}_{{\rm{Li}}}^{-}}$ decreases, while ${\rm{\Delta }}{E}_{{\rm{d}}}^{{{\rm{Li}}}^{+}}$ increases. At this range of Δ*μ*_Li_ the Fermi level shifts toward the valence band maximum leading to the increase of concentration of holes. Under this condition, the ionic conductivity takes place via formation and diffusion of ${{\rm{V}}}_{{\rm{Li}}}^{-}$. The calculated value of diffusion barrier for ${{\rm{V}}}_{{\rm{Li}}}^{-}$ and Li^+^ from DFT-NEB calculations^23^ are ${\rm{\Delta }}{E}_{{\rm{b}}}^{{V}_{{\rm{Li}}}^{-}}=0.27$ eV and ${\rm{\Delta }}{E}_{{\rm{b}}}^{{{\rm{Li}}}^{+}}=0.45$ eV, respectively. The activation energy of Li transport via the Frenkel mechanism is ${\rm{\Delta }}{E}_{{\rm{d}}}^{{V}_{{\rm{Li}}}^{-}}+{\rm{\Delta }}{E}_{{\rm{d}}}^{{{\rm{Li}}}^{+}}+{\rm{\Delta }}{E}_{{\rm{b}}}^{{V}_{{\rm{Li}}}^{-}}=1.8$ eV for −2.0 eV < Δ*μ*_Li_ ≤ 0 eV. However, the minimum value of activation energy is for the creation and diffusion of Li vacancy: ${\rm{\Delta }}{E}_{{\rm{d}}}^{{V}_{{\rm{Li}}}^{-}}+{\rm{\Delta }}{E}_{{\rm{b}}}^{{V}_{{\rm{Li}}}^{-}}=0.95$ eV at Δ*μ*_Li_ = −2.3 eV. Afterwards, we calculated the concentration of Li vacancy as function of chemical potential of Li (Fig. 3). We can distinguish two regimes for the concentration of Li: (i) low concentration regime for −2.0 eV < Δ*μ*_Li_ ≤ 0 eV with c′ = 2.8 × 10^9^ cm^−3^ and (ii) high concentration regime for −2.3 eV ≤ Δ*μ*_Li_ ≤ −2.0 eV with maximum c = 3.5 × 10^11^ cm^−3^. To calculate conductivity as function of temperature we will consider c′ and c.

**Figure 4:**
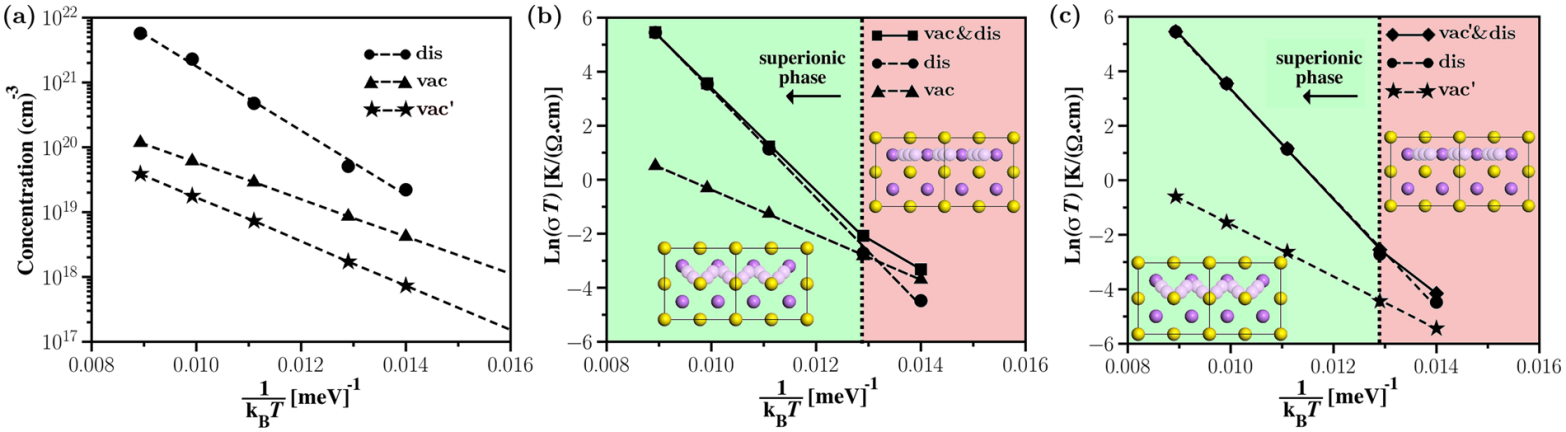
(**a**) Concentration of Li vacancy as function of temperature inverse calculated by eqs (1) and (2) for −2.3 eV ≤ Δ*μ*_Li_ ≤ −2.0 eV (vac) and −2.0 eV < Δ*μ*_Li_ ≤ 0 eV (vac′) as well as that of interstitial Li calculated by eq. (3) (dis). Li ion conductivities as function of temperature inverse for (**b**) vac&dis and (**c**) vac′&dis.

Figure 4 illustrates calculated concentration of ${V}_{{\rm{Li}}}^{-}$, namely (*n*_vac_), at Δ*μ*_Li_ = −2.3 eV at different temperatures using eq. (2). To estimate the concentration of Li at 4b sites in the superionic phase we used the detailed balance condition

3 $${n}_{{\rm{dis}}}=\frac{{n}_{{\rm{Li}}\mathrm{(8}c)}}{1+\frac{{\tau }_{8c}}{{\tau }_{4b}}},$$

where *n*_Li(8c)_ is the concentration of regular sites (4.27 × 10^22^ cm^−3^ for the theoretical lattice constant of 5.72 Å). *τ*_4*b*_ and *τ*_8*c*_ are the residence time for Li at 4b and 8c sites during AIMD simulations between 10 ps and 50 ps. The calculated values of *n*_dis_ for *T* = 830, 900, 1050, 1170 and 1300 K are illustrated in Fig. 4. We find that at 1300 K the concentration of 4b defect in the superionic phase is 48.6 times larger than the maximum possible concentration of ${V}_{{\rm{Li}}}^{-}$ (at Δ*μ*_Li_ = −2.3 eV) in poor-ionic conductor phase of Li_2_S.

Finally, the ionic conductivity as function of temperature is determined by

4 $${\sigma }_{{\rm{t}}ot}={\sigma }_{{\rm{v}}ac}+{\sigma }_{{\rm{d}}is}=\frac{{q}^{2}{F}^{2}}{{\rm{R}}T}[{n}_{{\rm{v}}ac}{D}_{{\rm{v}}ac}+{n}_{{\rm{d}}is}{D}_{{\rm{d}}is}]\mathrm{.}$$

where *q*, *F*, *R*, and *T* are charge of the carrier, Faraday constant, gas constant, and temperature, respectively. Calculated *σ* as function of temperature for two different concentration regimes is illustrated in Fig. 4. We find a clear superionic phase transition approximately at 900 K in the case of high concentration regime, which is in fair agreement with the Neutron scattering measurements by Altorfer *et al*.^22^ and Buehrer *et al*.^21^ showing that the superionic phase transition in Li_2_S takes place near 800 K and 900 K, respectively. Since phase transition at 900 K in the case of low–concentration regime is not very apparent, we will not discuss this case further. According to the Arrhenius plot of diffusion coefficients (see Fig. 1) the superionic phase transition starts at *T* = 1050 K, which is larger than *T* = 900 K at which disorder starts to form according to Figs 2 and 4. The value of *σ* at 300 K (9.43 × 10^−17^ S/cm) is very small, which is in agreement with the general belief that the value of *σ* for Li_2_S under ambient condition is very low. We are not aware of any experimental study measuring *σ* at 300 K.

The calculated value of *σ* at 1170 K is 3.02 × 10^−2^ S/cm, which is 4.2 times smaller than the experimental value of ≈1.27 × 10^−1^ S/cm (estimated from the conductivity versus temperature curve in ref. 22). The difference between experimental and theoretical values of *σ* might be because of (i) the underestimation of *n*_dis_ (needed for the calculation of *σ* in eq. (4)), which is due to the computational limitation for the AIMD simulations, as well as (ii) computational and experimental uncertainties. Finally, we find that the calculated values of *σ*_dis_/*σ*_vac_ at 900 K, 1170 K and 1300 K are 1.1, 47.1, and 138.9 respectively, showing that the enhancement of ionic conductivity in superionic regime is between one and two orders of magnitude depending on temperature.

In summary, we have combined AIMD and DFT calculations with thermodynamic and kinetic considerations to calculate diffusion coefficient, defect concentration, and ionic conductivity of Li_2_S as function of temperature. We find that Li ion transport at low temperatures (e.g. *T* = 830 K) occurs via Li vacancy hopping between regular Li sites. At and above the temperature of superionic phase transition, namely *T* = 900 K, Li ion transport takes place via both Li vacancy hopping between regular Li sites and Li hopping between regular and interstitial sites. At higher temperatures the latter mechanism becomes dominant. The increase in the concentration of interstitial Li plays the dominant role in the superionic behavior. For this reason although the calculated Arrhenius plot shows that the transition temperature to the superionic state is 1050 K, the calculated ionic conductivity shows a phase transition at 900 K, which is in agreement with experimental measurements. The presented approach in this work can be used to study superionic phase transition in other ionic crystals.

## Methods

### DFT Calculations

The DFT calculations were performed using the projector-augmented plane-wave code VASP^25–28^. The bulk Li_2_S was modelled by 3 × 3 × 3 super cells with 2 × 2 × 2 Monkhorst-Pack *k*-point mesh with an energy cutoff of 300 eV. We have calculated the electronic and atomic structures as well as defect formation energies using the generalized gradient approximation (GGA) exchange-correlation functional proposed by Perdew, Burke, and Ernzerhof (PBE)^29^.

### AIMD Simulations

The diffusion pathway and diffusion coefficient were evaluated using the *ab initio* MD (AIMD) calculations (implemented in VASP). AIMD calculations were performed in the canonical (NVT) ensemble with time steps of 1 fs. Bulk Li_2_S was modelled by 2 × 2 × 2 super cells with 4 × 4 × 4 Monkhorst-Pack *k*-point mesh with an energy cutoff of 360 eV. Mean Square Displacements (MSD) is obtained through the following equation:

5 $${\rm{MSD}}(\tau )=\frac{1}{{N}_{atom}}\frac{1}{{N}_{step}-\tau }\times {\sum }_{j=1}^{{N}_{atom}}{\sum }_{i=1}^{{N}_{step}-\tau }{|{\vec{r}}_{j}({t}_{i}+\tau )-{\vec{r}}_{j}({t}_{i})|}^{2},$$

where *τ* is lag time, *N*_*atom*_ and *N*_*step*_ are the number of diffusing Li ions and number of AIMD time steps (in our work 40000). Diffusion coefficient *D* is calculated using the Einstein relation:

6 $$D=\mathop{\mathrm{lim}}\limits_{\tau \to \infty }\frac{{\rm{MSD}}(\tau )}{6\tau }\mathrm{.}$$
